# The Development and Validation of Food Atlas for Portion Size Estimation in the Balkan Region

**DOI:** 10.3389/fnut.2018.00078

**Published:** 2018-09-13

**Authors:** Marina Nikolić, Jelena Milešević, Milica Zeković, Mirjana Gurinović, Marija Glibetić

**Affiliations:** ^1^Center of Research Excellence in Nutrition and Metabolism, Institute for Medical Research, University of Belgrade, Belgrade, Serbia; ^2^Capacity Development Network in Nutrition in Central and Eastern Europe (CAPNUTRA), Belgrade, Serbia

**Keywords:** food picture book, portion size, nutritional assessment, validation, food consumption survey

## Abstract

Assessment of portion sizes is an important factor for the accuracy of food consumption surveys. The objective of this study was to develop and validate a food atlas of commonly consumed foods in the Balkan region in order to improve the accuracy of portion size estimation for food consumption surveys. A list of 135 foods and their portion sizes was based on previously conducted food consumption surveys in this region. Food was cooked, measured and served in three or four portion sizes right before being photographed. A validation study was conducted through the visual perception method. Without receiving training on usage of the food picture book, participants were asked to evaluate two portion sizes of 20 selected foods by comparison with a photo series of each food. Portion sizes were evaluated by 18 nutrition professionals and 17 lay individuals who had no nutritional education. Mean differences and the standard deviations of the mean differences (SD) between the portions estimated by each participant and the served portion were calculated. The percentages of participants who selected the correct, adjacent or distant portion size also were calculated. The number of food items that were quantified within the predefined acceptable range (i.e., mean difference < ∣ 0·7 ∣ and *SD* < 1) was 16 (80%) among lay individuals and 17 (85%) among nutritional professionals. Among 16 photo series that were assessed as “acceptable,” the percentage of all participants, who selected the correct picture, was between 44.3 and 82.9%, with an average of 60.2%. Only three foods were assessed correctly by <50% participants. The percentage of participants who selected the correct or adjacent serving size was above 98% for both lay and professional evaluators. This is the first food atlas containing representative foods and recipes commonly consumed in the Balkan region. However, further adjustments of the methodology should include larger number of food items to be tested, involvement of more participants and provision of training for the users of the food atlas. This food atlas could be used in food consumption surveys in the Balkan region after further testing and validation.

## Introduction

The development of much needed research infrastructure for nutrition in the Balkan region requires designing research tools that are standardized and validated to ensure comparable results in an international context (1). In the past decade, different elements of research infrastructure were developed: a food composition database (2), dietary software for data collection and nutrient calculation (3), and nutritional planning and dietary assessment software with incorporated nutritional recommendations (4). All of these are prerequisite for conducting food consumption surveys and other nutritional research.

However, one of the biggest challenges in food consumption data collection is estimation of the amount of consumed foods. The most accurate method for measuring food intake is weighing foods before and after consumption, known as “weighed food intake method.” This method has many disadvantages: it is costly, time-consuming, can influence consumption, and cause participant fatigue. Its application becomes increasingly difficult in epidemiological studies, e.g., in circumstances where scales might not be available. Thus, other methods have been designed to help individuals estimate the amount of food consumed. For accurately completing food frequency questionnaires or other types of food consumption surveys, pictorial food portion guides that represent different portion sizes have been developed (5). Portion guides have been shown to be a valid method to help respondents estimate the amount of consumed foods (6, 7). These improve the accuracy of measurements, particularly in large scale studies in the general population (5, 8). A food atlas, a pictorial guide of foods with different food portion sizes, contains photos of the foods that are representative of the national/regional diet of the population, with the ranges of portion sizes usually consumed.

The objective of this study is to present the process of development and validation of a food atlas containing photos of typical foods and most consumed dishes in the Balkan region. The food atlas was tested and validated for its accuracy among nutritional professionals and lay individuals. The atlas is integrated in the regional food composition database (2) and dietary assessment tool - Diet Assess and Plan (DAP) (9), and also printed in hardcopy versions for fieldwork for future dietary surveys.

## Methodology

### Subjects and study design

In order to define a format for the food atlas for the Balkan region numerous food atlases from other countries/regions and scientific publications on their development and validation were reviewed (10–19). The development of the food atlas included three steps: selection of foods and recipes, determination of portion sizes, and photographing, followed by coding of photos and codebook creation. After food atlas development, a validation study was performed using the perception method as previously described (20). The ability of study participants to assess food portion sizes was tested by choosing correct food portion size from a photo series after exposure to real food portions (8, 15, 20). This survey was approved by the ethical committee of the Institute for Medical Research, University of Belgrade (No. EO123/2017).

### Selection of foods and recipes

Based on previously conducted food consumption surveys in the Balkan region the most consumed foods and recipes were selected for the food atlas (21–24). Furthermore, to address local dietary patterns and improve cultural competency of the atlas, additional dishes from traditional cookbooks, and restaurant menus were included. A final list of 125 items was reviewed and approved by a panel of five experts in nutrition and dietary intake assessment. The panel consisted of two senior researchers in the field of nutrition and three *post-doc* researchers, experts in food consumption surveys. The choice of food items within food groups corresponds to common dishes or food types in the Balkan dietary pattern. The food atlas might be use for the assessment of other food items and dishes not included in the food atlas but of similar appearance/shape and texture or composition like ones presented in the photos. A list of selected food items for children was created based on previous surveys and a food list provided by the EU Menu protocol for development of food atlases for children (25). Dishes consumed in a home environment are usually served using different ladle or other utensil sizes, while beverages are often consumed directly from the original packaging such as cans, bottles or paperboards. Thus, 5 utensils, 6 different types of liquid packaging, and baby bottles are represented. The draft version of the food atlas was used in a pilot food consumption survey in Serbia, Montenegro, and Bosnia and Herzegovina for the assessment of food portion sizes reported in 24 h recalls among adults and food diaries among children. After the pilot study, field workers highlighted 10 food items that were consumed by participants, but were not present in the food atlas and could not be assessed by any other available photo. Therefore, additional photos with different portions for the selected 10 food items were added to the food atlas. The final version of the food atlas contains 135 foods, 5 utensils, and 6 liquid packaging types. The structure of the final version, based on food group classification, is presented in Table 1.

**Table 1:** Number of selected food items and recipes present in the food picture book according to food group.

**Food group**	**Simple foods (*n*)**	**Recipes dishes**	**Total (*n*)**
Milk and milk products	6	–	6
Eggs	–	4	4
Meat or meat products	10	10	20
Seafood or related products	–	3	3
Grain or grain products	13	16	29
Nuts, seeds or kernel products	1	–	1
Vegetables or vegetable products	19	16	35
Fruit or fruit products	16	–	16
Sugar and sugar products/sweets	1	10	11
Non-milk beverages	2	–	2
Miscellaneous food products	3	5	8
Utensils	–	–	5
Packaging	–	–	6

### Selection of portion sizes

**Figure 1:**
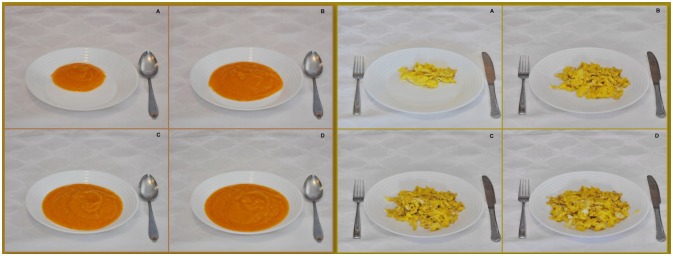
Photo series of carrot soup **(left)** and scrambled eggs **(right)**.

Medium portion sizes for all selected foods and recipes were defined based on the average portion from previous food consumption surveys in the Balkan region (22–24). A coefficient equal to 1 was assigned to the medium portion size, while the small portion was calculated by multiplying the medium portion size by 0.5. The large portion size was calculated by multiplying the medium one by 1.5, while the extra-large was calculated by multiplying the large portion by 1.5. Portion sizes for children were adopted from the EU Menu protocol for the development of food atlases for children (25). Photo series of selected food items and recipes consist of three or four photos per series, depicting a range of portion sizes in increasing order as shown in Figure 1. This way of presenting a photo series was suggested by the EU Menu methodology proposed by EFSA as the most commonly used (25). A few standardized bakery products (e.g., doughnuts and bread rolls) and barbeque meals (e.g., ćevapčići and standard traditional sausage) are presented as a single photo. Food items that could be served in diverse ways were presented in more than 4 photos like sliced or diced cucumber, whole or sliced tomato, etc. A unique numerical identification code was assigned to each food item. Photographs within series were labeled with uppercase letters and arranged alphabetically to assign a gradual increase in portion size. In order to avoid response bias and misreporting, actual weights of portions were not indicated in conjunction with photographs, but at the end in a separate codebook.

### Food preparation, presentation, and photographing

Food items and recipes were prepared and served by a professional cook who was provided with detailed guidance on how to prepare, measure, and serve food for photographing. Prepared food was measured on a digital kitchen scale Beurer KS 19 with a precision of 1 g and a maximum capacity of 5 kg, calibrated for the serving plate weight. Food items were served on a white table cloth and on a white standard shallow or deep plate (diameter = 24 cm) or small shallow plate (diameter = 20 cm) or small white bowl (diameter = 18 cm). For the assessment of beverages several types of glassware are presented. Four types of different glassware for water and other non-alcoholic beverages are shown to depict different volumes in different glass shapes (150, 250 ml-two different shapes, 330 ml). Wine and shot glasses are captured with three different levels of liquid amounts to help in the assessment of alcoholic beverage consumption. Cutlery was always placed adjacent to the plate for easier perception of plate size. All selected food items were captured by a professional photographer under standard lighting conditions using a digital camera Nikon D80. All the photos were taken in identical conditions: angle of 45 degrees and the distance of 55 cm above the plate with a white background and aperture between 11–16 and focal length of 100 mm. The camera was always mounted on a tripod above the plate at eye level of a seated person of average height.

### Validation study

A validation study was conducted through the visual perception of food portions by comparison with a photo series of shown food (8, 15, 20, 26, 27). The aim of this survey was to validate the food atlas for the usage by nutrition professionals (28) and people without any nutritional background (lay individuals) (20). The rationale was to test its relevance, accuracy and applicability to both groups of users, i.e., those who are more trained to estimate portions sizes and those who are less trained but represent prospective respondents. Therefore, the study sample involved two groups of volunteers: 18 professionals with documented educational background/expertise in nutrition (average age: 46.4 ± 9.3 years) and 17 subjects without any formal training or professional experience in the field (average age: 36.4 ± 7.2 years). Verbal informed consent was obtained from all participants. Females were overrepresented as they expressed more interest in the survey. Eight out of 35 participants were mothers of children younger than 10 years. The validation session took place in the hotel meeting room during the Capacity Development in Nutrition Network—CAPNUTRA Symposium, Capacity development in dietary intake survey - harmonization with EU MENU methodology regional training for DIET ASSESS & PLAN (DAP) application and implementation in the Balkan region (www.capnutra.org). The training involved 35 participants from five countries, including Bosnia and Herzegovina, Montenegro, Former Yugoslav Republic of Macedonia (FYROM), Italy and Serbia. Food was prepared, measured on a digital kitchen scale and served by the hotel's staff guided by nutrition professionals from the CAPNUTRA team.

The study was designed to have 20 photo series evaluated through 40 food portions (two different randomly selected portions of each food item). In this way 50% of portion sizes were evaluated as suggested by the EU Menu protocol for the validation of food atlases (20). A list of evaluated food items is presented in Table 2. Food items were placed on the table so that two different portions of the same food item were not next to nor close to each other. Corresponding coded photo series of three or four photos representing different portion sizes in increasing order, just as in the food atlas, were placed above each plate. Printed evaluation forms, with the list of foods in the order of serving, were provided to all participants 2 h before the session, with both written and oral instructions on how to fill them. In addition, participants received a clear verbal explanation on how to approach the evaluation and assess the sizes of served portions. However, the participants did not receive training on the assessment of portion sizes using the food atlas, as we wanted to get comprehensive feedback on the applicability and accuracy of the tool. Members of the CAPNUTRA team were answering all of the participants' questions related to the evaluation to clarify any ambiguities. Communication between participants was not allowed during the session. All participants evaluated both served portion sizes of each food and wrote the selected code from the photo series next to the name of the food in the evaluation form. In this way each food (photo series) was evaluated 70 times. The utensils and packaging were not tested within the validation study, as their volumes are standardized.

**Table 2:** List of evaluated foods and recipes with mean and standard deviation (SD) of the difference between the estimated portion number minus the actual portion number; Spearman's coefficients of correlation (*r*) between the estimated portion number and the actual portion number.

	**Lay individuals**			**Nutrition professionals**			**Total**		
	**Mean difference**	***SD***	***r***	**Mean difference**	***SD***	***r***	**Mean difference**	***SD***	***r***
Pasta	0.00	0.70	0.73^**^	0.53	0.56	0.95^**^	0.27	0.68	0.76^**^
Rice with vegetables	0.38	0.49	0.82^**^	0.42	0.50	0.70^**^	0.40	0.49	0.76^**^
Baked beans	−0.03	0.46	0.65^**^	0.14	0.42	0.69^**^	0.06	0.45	0.66^**^
Banana, mashed	−0.68	0.47	0.72^**^	−0.06	0.41	0.72^**^	−0.36	0.54	0.63^**^
Scrambled eggs	−0.29	0.58	0.87^**^	−0.25	0.55	0.87^**^	−0.27	0.56	0.87^**^
Cake	0.85	0.61	0.72^**^	0.58	0.50	0.84^**^	0.71	0.57	0.77^**^
Cabbage grated	−0.94	0.34	0.82^**^	−1.28	0.45	0.54^**^	−1.11	0.44	0.65^**^
Banana, whole unpeeled	0.47	0.51	0.71^**^	0.64	0.54	0.62^**^	0.56	0.53	0.66^**^
Soup	−0.06	0.60	0.50^**^	−0.50	0.51	0.75^**^	−0.29	0.59	0.61^**^
Ground biscuit	−0.18	0.76	0.69^**^	−0.39	0.60	0.84^**^	−0.29	0.68	0.77^**^
Lettuce	1.35	0.49	0.95^**^	1.03	0.38	0.95^**^	1.19	0.46	0.94^**^
Potato, mashed	0.62	0.55	0.66^**^	−0.17	0.56	0.55^**^	0.21	0.68	0.51^**^
Grape	0.56	0.50	0.84^**^	0.56	0.50	0.71^**^	0.56	0.50	0.78^**^
French fries	0.53	0.56	0.75^**^	0.67	0.72	0.86^**^	0.60	0.65	0.81^**^
Walnuts	−0.26	0.51	0.51^**^	−0.11	0.32	0.80^**^	−0.19	0.43	0.65^**^
Cream	−0.21	0.69	0.37^*^	0.08	0.50	0.72^**^	−0.06	0.61	0.55^**^
Carrot, grated	−1.15	0.78	0.74^**^	−1.42	0.60	0.89^**^	−1.29	0.70	0.80^**^
Ham	0.32	0.47	0.91^**^	0.39	0.55	0.82^**^	0.36	0.51	0.86^**^
Apple	0.18	0.58	0.67^**^	0.50	0.56	0.42^*^	0.34	0.59	0.53^**^
Spinach	0.14	0.49	0.76^**^	0.05	0.47	0.77^**^	0.10	0.45	0.63^**^

Statistically significant correlations are indicated with:

* *p < 0.05*,

** *p < 0.01*.

*Shaded food items are those assessed out of the acceptable range*.

### Statistical analysis

All pictures were numbered from 1, for the smallest portion, to 4, for the largest portion. Mean difference and standard deviations were calculated between the portion number estimated by participant and served portion number, for data obtained from nutrition professionals, lay individuals and all participants together reflecting the total sample. The limit of bias of the mean difference is ~0.7 calculated as 99.999% CI of the estimation error using normal distribution. Standard deviation of the mean difference <1 was used since a random error of choosing adjacent photo (±1) was considered as acceptable. Choosing the adjacent photo could not lead to significant over- or under- reporting of energy intake since this misreporting account for <10% of the average daily energy intake for the most energy—dense food items such as pasta. Moreover, it is expected that under—and overestimated portion sizes, by choosing adjacent photo, of different foods would attenuate the error. The high percentage of CI was chosen because it gives a wider range of acceptance for variables where the mean value is not significantly different from 0 (15). Spearman correlation coefficients between the chosen and correct number of the photo were calculated for both groups separately and for the total sample. Percentage of participant choosing the correct, adjacent (±1) or distant (>1, <-1) number of photo was calculated and chi square test was applied to check for differences in assessment between nutrition professionals and lay public group. Results are considered significant at *p* < 0.05. All analyses were performed using the IBM SPSS software package 20.0.

## Results

Means and standard deviations of the differences between the estimated picture number minus the correct portion size number and Spearman correlation coefficients between the estimated picture number and the actual portion number are summarized in Table 2. The number of food items that were quantified within the predefined acceptable range (i.e., mean difference < ∣ 0·7 ∣ and *SD* < 1) was 16 (80%) among lay individuals and 17(85%) among nutritional professionals. On the other hand, nutrition professionals quantified 29 (72.5%) portions within the acceptable range, while lay individuals correctly assessed 26 portions (65%). However, chi-square test has shown that there were no statistically significant differences between nutrition professionals and lay individuals regarding the number of foods, quantified within the acceptable range, (*p* = 0.576). Spearman correlation coefficients between the estimated picture number and the correct picture number were between 0.5 and 0.95 and significant at 0.01 level among both groups and on the total sample with exception of cream for the lay public group (*r* = 0.37, *p* = 0.031) and apple among nutrition professionals (*r* = 0.42, *p* = 0.01). Sixteen out of twenty selected photo series performed well, while four food items were assessed correctly by < 35% participants and with mean difference > ∣ 0.7 ∣. In general, the assessment of small and medium portions was better (64.9 and 45.9% correct answers, respectively), than assessment of large and extra-large portion (41–43.8% correct answer). Participants were more likely to overestimate small and medium portion and underestimate large and extra-large portion sizes as presented in Table 3. The percentages of participants choosing the correct, adjacent or distant number of a picture when comparing shown food items with photo series are presented in Table 4. Among 16 photo series that were assessed as “acceptable,” the food items that were correctly assessed by the lowest percentage of participants are: grape (44.3%) among the total sample, raw banana (38.9%) among nutrition professionals, and mashed banana (32.4%) among lay public. The food items that were correctly assessed by majority of participants are: baked beans among the total sample and lay public (82.9 and 82.4%, respectively) and walnuts (88.9%) among nutrition professionals. The percentage of participants who selected correct or adjacent number of photo series was above 98% for both groups and for the total sample. Distant photo in the series was chosen by 1.4–8.6% participants on total sample. Mashed banana, ground biscuit and walnuts were better assessed by nutrition professionals than by the lay public group (*p* < 0.001, *p* = 0.019, *p* = 0.03, respectively), while there were no significant differences among other food items. Four picture series that didn't performed well were cake, grated fresh cabbage, lettuce and grated carrot (shaded in gray in Tables 2, 4). These food items were assessed correctly by only 2.9–34.3% of participants from the total sample, while the mean difference indicated an overestimation for cake and lettuce with mean difference of 0.71 and 1.19 respectively and underestimation of grated cabbage (−1.11) and grated carrot (−1.29). Distant picture was chosen by 5.7% of participants for cake, 15.7% for grated cabbage, 21.4% for lettuce, and 40% for grated carrot.

**Table 3:** Proportion of correct responses and direction of errors for the estimation of portion sizes in the total sample.

**Portion size**	**Correct (%)**	**Underestimated^a^ (%)**	**Overestimated^b^ (%)**
Small	64.9	0	35.1
Medium	45.9	19.7	34.4
Large	41.0	32.4	26.7
Extra large	43.8	56.2	0
All portions	49.4	20.4	30.2

a *Underestimated—selected portion estimate was lower than served*.

b *Overestimated—selected portion was bigger than the served one*.

**Table 4:** Percentages of participants choosing the correct, adjacent, or distant picture when comparing shown food items with the photo series.

	**Lay individuals (*****n*** = **17)**							**Nutrition professionals (*****n*** = **18)**							**Total (*****n*** = **35)**							
	**Correct (%)**	**Adjacent (%)**			**Distant (%)**			**Correct (%)**	**Adjacent (%)**			**Distant (%)**			**Correct (%)**	**Adjacent (%)**			**Distant (%)**			
	**0**	**−1**	**1**	**Total**	**< −1**	**>1**	**Total**	**0**	**−1**	**1**	**Total**	**< −1**	**>1**	**Total**	**0**	**−1**	**1**	**Total**	**< −1**	**>1**	**Total**	***p*-value**
Pasta	52.9	23.5	23.5	47	0	0	0	50	0	47.2	47.2	0	2.8	2.8	51.4	11.4	35.7	47.1	0	1.4	1.4	
Rice and vegetable	61.8	0	38.2	38.2	0	0	0	58.3	0	41.7	41.7	0	0	0	60	0	40	40	0	0	0	
Baked beans	79.4	11.8	8.8	20.6	0	0	0	80.6	2.8	16.7	19.5	0	0	0	80	7.1	12.9	20	0	0	0	
Banana, mashed	32.4	67.6	0	67.6	0	0	0	83.3	11.1	5.6	16.7	0	0	0	58.6	38.6	2.9	41.5	0	0	0	< 0.001
Scrambled eggs	58.8	35.3	5.9	41.2	0	0	0	63.9	32.9	5.7	38.6	0	0	0	61.4	32.9	5.7	38.60	0	0	0	
Cake	26.5	0	61.8	61.8	0	11.8	11.8	41.7	0	58.3	58.3	0	0	0	34.3	0	60	60	0	5.7	5.7	
Cabbage, grated	8.8	88.2	0	88.2	2.9	0	2.9	0	72.2	0	72.2	27.8	0	27.8	4.3	80	0	80	15.7	0	15.7	0.005
Banana, whole unpeeled	52.9	0	47.1	47.1	0	0	0	38.9	0	58.3	58.3	0	2.8	2.8	45.7	0	52.9	52.9	0	1.4	1.4	
Soup	64.7	20.6	14.7	35.3	0	0	0	50	50	0	50	0	0	0	57.1	35.7	7.1	42.8	0	0	0	
Ground biscuit	41.2	38.2	20.6	58.8	0	0	0	66.7	27.8	0	27.8	5.6	0	5.6	54.3	32.9	10	42.9	2.9	0	2.9	0.019
Lettuce	0	0	64.7	64.7	0	35.3	35.3	5.6	0	75.7	75.7	0	8.3	8.3	2.9	0	75.7	75.7	0	21.4	21.4	0.012
Potato, mashed	41.2	0	55.9	55.9	0	2.9	2.9	66.7	25	8.3	33.3	0	0	0	54.3	12.9	31.4	44.3	0	1.4	1.4	
Grape	44.1	0	55.9	55.9	0	0	0	44.4	0	55.6	55.6	0	0	0	44.3	0	55.7	55.7	0	0	0	
French fries	50	0	47.1	47.1	0	2.9	2.9	47.2	0	38.9	38.9	0	13.9	13.9	48.6	0	42.9	42.9	0	8.6	8.6	
Walnuts	67.6	29.4	2.9	32.3	0	0	0	88.9	11.1	0	11.1	0	0	0	78.6	20	1.4	21.4	0	0	0	0.03
Cream	58.8	32.4	5.9	38.3	0	2.9	2.9	75	8.3	16.7	25	0	0	0	67.1	20	11.4	31.4	0	1.4	1.4	
Carrot, grated	20.6	47.1	0	47.1	32.3	0	32.3	5.6	47.2	0	47.2	47.2	0	47.2	12.9	47.1	0	47.1	40	0	40	
Ham	67.6	0	32.4	32.4	0	0	0	63.9	0	33.3	33.3	0	2.8	2.8	65.7	0	32.9	32.9	0	1.4	1.4	
Apple	64.7	8.8	26.5	35.3	0	0	0	44.4	2.8	52.8	55.6	0	0	0	54.3	5.7	40	45.7	0	0	0	
Spinach	82.4	0	14.7	14.7	0	2.9	2.9	83.3	0	16.7	16.7	0	0	0	82.9	0	15.7	15.7	0	1.4	1.4	

*Shaded food items are those that were out of the acceptable range*.

## Discussion

We conducted this study to develop and evaluate the first food atlas in the Balkan region, based on previous knowledge of the dietary habits and commonly consumed food among adults in this region. In the evaluation process, we applied the prospective method, which was previously used in similar studies. The results of our study are in accordance with the evaluation of food atlases for children in three European countries, where using similar methodologies, 50% of parents selected the correct photo in the food atlas (15). In addition, a study of Finnish adults showed that between 43 and 65% of participants chose the correct photo (27) while in our study the correct photo was chosen by 60.3% participants in average (44.3–82.9%). However, the correct or adjacent photo was chosen by more than 98% of participants in our study while in Greek adults more than 90% of participants chose the correct or adjacent photo (8), with even lower percentage found among Belgium adolescents (71%) (26).

Two studies reported a Spearman correlation coefficient of 0.5–0.9 between the number of shown and chosen photo (15, 26) which is in accordance with our results. In our study, challenges are recognized with some specific food items such as cake. Trolle et al. found that it is crucial for the shape of the cake to be as similar as possible as on the shown photo, which is not always possible due to the many existing types of cakes (15). How the cake is served was also recognized as an important factor e.g., whether the piece of cake was placed vertically on the plate or laid down. Lettuce and mixed salads were found challenging by Trolle et al. too, as a shapeless food (like grated cabbage and carrots in our study) due to difficult presentation in exactly the same way as on the photos (15). Similar issues were recognized in a recent study by Subar et al. (29).

However, the methodology of the present study could be improved by several aspects. Error in perception due an inadequate photo would have a much larger impact on energy and macronutrient intake estimation when high caloric foods are misrepresented and inadequately assessed than with foods such as lettuce, cabbage, or carrots. For instance, choosing a distant photo for cake would lead to the under or overestimation of energy intake of ~250 kcal, while making the same error with lettuce would result in 11 kcal. Thus, the methodology could be improved by providing more photos of these particular foods in different shapes and forms. In addition, the whole experience in this study has shown us that training for users on how to appropriately employ the food atlas in contact with correspondents, during the food consumption surveys, would certainly increase the accuracy of their assessments. Particularly, the training could prepare users to assist respondents in finding appropriate alternatives when some food they ate is missing in the food atlas and help them recall the food portion size without influencing the estimation.

The strength of this study is that this is the first food atlas to be developed in the Balkan region containing the most consumed food items and dishes and validated according to approved methodology. These photos are integrated and connected to appropriate foods data in the regional FCDB (2), which allows faster data collection and further processing in DAP software (9). Furthermore, hardcopy version of the Food Atlas has been used as a portion size estimation aid in food consumption survey based on EU Methodology in the Balkan region (Serbia, Bosnia and Herzegovina, Montenegro, and Macedonia- with translation to Macedonian).

The limitation of this study is related to the limited number of food portions tested and relatively small number of assessors, in both groups. In future studies larger number of food items and assessors should be engaged for the validation of the tool. Particularly, the number of male participants is critically small in both groups, which might question the adequacy of future estimations in this population group. Statistical analysis has shown that there were more participants who tended to underestimate large and extra-large portions than those who tended to overestimate small and medium portion. Thus, usage of this tool could lead to the underreporting of energy and nutrient intake. Analysis in the future should include the effects of factors such as gender and weight status on the assessment outcome.

## Conclusion

The accuracy of portion size estimates is essential in the assessment of food consumption. In order to provide information of the portion sizes of commonly consumed food items and traditional dishes, a food atlas was developed with food portions presented in photos. This is the first food atlas of the most representative diet in the Balkan region. Food items, challenging for assessment, were recognized in the applied validation process. However, further adjustments of the methodology should include larger number of food items to be tested, involvement of more participants (both, professionals and lay individuals) and provision of training for the users of the food atlas. This food atlas could be used in food consumption surveys in the Balkan region after further testing and validation.

## Author contributions

MaG, MiG, and MN were responsible for the formulation of the research question and designing the study. MN, JM, and MZ were responsible for caring it out. MN was responsible for the data analysis. MN and JM drafted the manuscript. JM, MZ, MiG, and MaG edited the working versions of the manuscript and provided advice regarding interpretation. MN had primary responsibility for the final content. All authors read and approved the final manuscript.

### Conflict of interest statement

The authors declare that the research was conducted in the absence of any commercial or financial relationships that could be construed as a potential conflict of interest.
